# Low-cost and versatile integration of microwire electrodes and optical waveguides into silicone elastomeric devices using modified xurographic methods

**DOI:** 10.1038/micronano.2017.40

**Published:** 2017-10-09

**Authors:** Juncong Liu, James B Mahony, Ponnambalam Ravi Selvaganapathy

**Affiliations:** 1 Department of Mechanical Engineering, McMaster University, Hamilton, Ontario L8S 4L7, Canada; 2 Department of Pathology and Molecular Medicine, McMaster University, Hamilton, Ontario L8S 4K1, Canada; 3 School of Biomedical Engineering, McMaster University, Hamilton, Ontario L8S4K1, Canada

**Keywords:** lab on a chip, microelectrode, polydimethylsiloxane (PDMS), xurography

## Abstract

Microelectrodes are used in microfluidic devices for a variety of purposes such as heating, applying electric fields, and electrochemical sensing. However, they are still manufactured by expensive deposition techniques such as sputtering or evaporation and patterned using photolithography methods. More recently, alternate methods including nanoparticle sintering and use of liquid metal flowing through microchannels have been used to fabricate microelectrodes. These methods are limited in the material choices or require post processing to be integrated into microchannels. Here we developed a low-cost and versatile method to integrate high-quality metal microwires into polydimethylsiloxane (PDMS) using xurography. The microwire integration process includes cutting slit pattern on PDMS substrate and subsequent writing metal microwires into the slit pattern using a specialized tip. Then the microwire-integrated PDMS was sealed/bonded using uncured PDMS prepolymer. This method enables integration of metal microwires of diameter as small as 15 μm into PDMS devices. Integration of multiple microwires with minimum spacing of 150 μm has also been demonstrated. The versatility of this method is demonstrated by the fabrication of metal microwire suspended in the middle of the microchannel, which is difficult to achieve using conventional electrode fabrication methods. This low-cost method avoids expensive clean room fabrication yet producing high-quality electrodes and can be used in a variety of microfluidic and MEMS applications.

## Introduction

Polydimethylsiloxane (PDMS) has been one of the most widely used materials for lab on chip (LOC) device prototyping and fabrication due to its remarkable properties. These properties include transparency, elasticity, oxygen-permeability, low surface energy, among others. The low surface energy facilitates reliable delamination of the PDMS cast structures from the mold, but also produces weak bonding between PDMS and any metal microstructures making it a challenge to integrate electrodes into PDMS devices. These electrodes are important functional elements of microfluidic devices used for electrochemical sensing, particle manipulation, sample concentration, mixing, separation, and others.

Physical vapor deposition (PVD), is one of the most common method for thin film electrode fabrication^1^. In this method, evaporated or sputtered vapor from the source metal is deposited on the substrate and patterned using photolithography. These thin electrodes are typically 10’s of nm to several micrometers thick and are of high quality and conductivity. However, they have long processing time, require high vacuum and expensive deposition equipment. Photolithographic definition of the electrode shapes and patterns further increase the cost and time. Alternatively, direct write methods using metal nanoparticle inks have been used. Traces of metal nanoparticles formed in this process can be sintered at low temperature due to melting point reduction. These nanoparticles are dissolved in solvent and deposited through thick film technologies including inkjet printing^2^ and screen printing^3^ and then sintered by heating^4^, laser writing^4^, or chemical sintering^5^. The cost and time required for these processes is much less than PVD but the resolution is around 100 μm (Ref. 6) with low geometrical uniformity and poor material properties^7^. The minimum thickness of electrodes formed by these processes is around several micrometers, which produces poor conformal sealing of bonded PDMS devices. In both PVD or particle sintering methods, the microelectrode deposition directly on PDMS require surface modification^8–10^ and the electrodes themselves are subjected to buckling and cracking due to the difference of thermomechanical properties. Alternatively, microelectrodes can be deposited on other substrates such as glass and plastics and are then bonded to PDMS^3,11^, which changes the channel properties such as elasticity, hydrophobicity, and gas permeability. Conductive nanoparticles can also be mixed with PDMS prepolymer to make it conductive^12,13^. The conductive polymer can be patterned by photolithography^14^, microcontact printing^15^ or screen printing^16^. These conductive polymer electrodes are flexible and can be integrated in 3D structure^13,17^. However, conductivity of these polymer electrodes is much lower than metal and photolithography is required to make the microelectrodes^18^. Low melting point metal have also been widely used to make electrodes in PDMS by injecting and casting them in specially designed microchannels. These metallic electrodes are highly conductive, flexible, and self-healing. By appropriately designing the microchannel, these electrodes can be insulated from the fluidic channel^19,20^ or exposed to the it^21,22^. It can also be designed to fill a dead-end channel^23^ and create 3D complex structures^24,25^. However, the microchannels used to define the electrode pattern are also fabricated by photolithography. Furthermore, these electrodes are of the same height as the fluidic channel and cannot pass through the microchannel without blocking it. Finally, the available choices of electrode materials are also limited^24^.

Microwires can also be integrated into PDMS devices as microelectrodes. These microwires are commercially available therefore no extra fabrication process is required. They are also well manufactured with small production tolerance in diameter (1–3% for diameter<100 μm)^26^, resistance (commonly ±3%)^27^ and other properties for specific applications^28^. These microwires could be as small as 4 μm and available in gold, silver, platinum, copper, palladium, Ni–Cr alloy to name but a few. Nonetheless, an efficient way to integrate these microwire into PDMS has not been developed yet. They are either fitted in specially designed microchannel^29,30^, fixed on the master mold before pouring PDMS^31,32^, or just manually inserted into the reservoir^33,34^. As the microchannels and fillisters are not able to hold the microwires very tightly, they can only be used to integrate straight microwire electrodes. In this paper, we introduce a method to integrate microwire electrodes into PDMS devices based on a modified xurography process. Xurography has been developed as a low-cost alternative to photolithography to fabricate channels in PDMS^35–37^ with low cost-cutting plotter. By incorporating electrode integration process into the cutting plotter, a low-cost and versatile PDMS LOC devices fabrication platform has been developed. The cutting plotter was used to pattern plastic sheets to form the master mold for PDMS micromolding, cut slits on cured PDMS surface and to write microwire electrodes into these patterned slits. The device bonding and sealing was completed by wet bonding. The microwire electrodes were integrated into PDMS devices with high flexibility in position and could also be suspended inside the microchannel with simple patterns. Several applications are shown to demonstrate the versatility of the modified xurographic method.

## Materials and methods

### Fabrication of PDMS microfluidic channel

**Figure 1:**
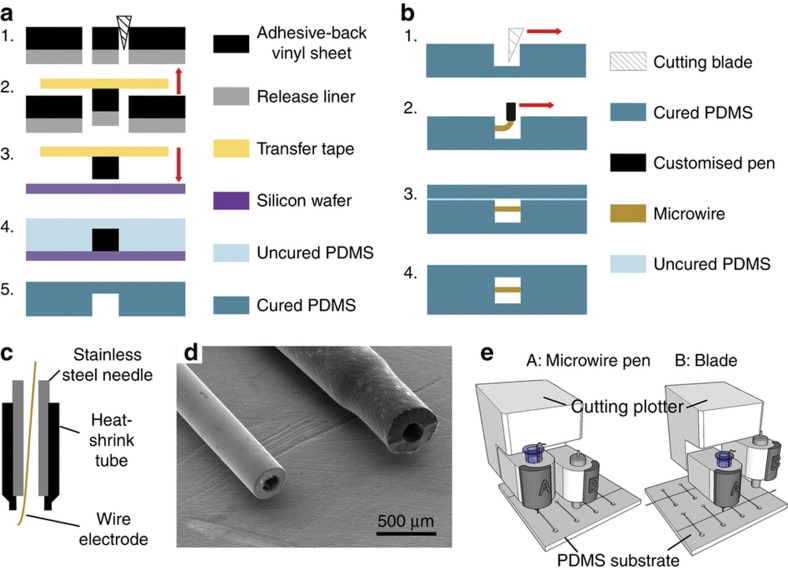
(**a**) Schematic of xurographic polydimethylsiloxane (PDMS) microchannel fabrication, 1—adhesive-back sheet is patterned by cutting plotter. 2—the pattern is peeled off from the cutting mat by a transfer tape. 3—The pattern is transferred to a silicon wafer as mold. 4—10:1 PDMS prepolymer is cast on to the mold. 5—the cast PDMS is cured and peeled off. (**b**) Schematic of xurographic metal microwire integration, 1—slits are cut on the PDMS using cutting plotter. 2—microwires are written into patterned slits 3—the device with integrated metal microwire is bonded to another PDMS surface using PDMS prepolymer wet bonding. 4—the two PDMS substrate with integrated metal microwire is bonded/sealed by curing the prepolymer. (**c**) Structure of the microwire pen. (**d**) Scanning micron microscopic image of the microwire pen tip without (left) and with (right) encapsulated heat-shrink tube. (**f**) Schematic of slit cutting and metal microwire writing completed with a low-cost multifunctional cutting plotter.

**Figure 2:**
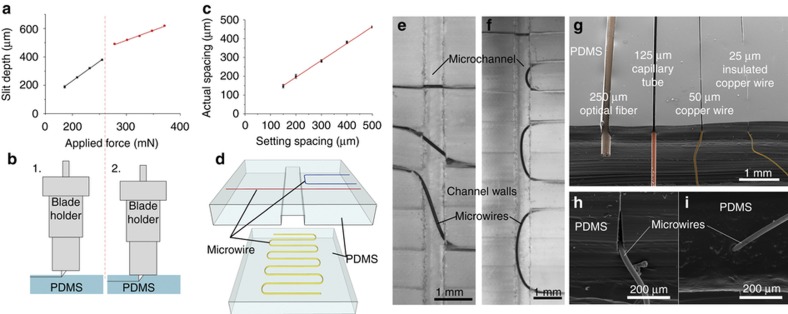
(**a**) Dependence of slit depth on the force applied during the cutting process (15 repeats). (**b**) Relationship between the PDMS substrate and the blade holder during cutting. 1—under low cutting force, the blade holder is not in touch with the PDMS substrate. 2—under high cutting force, the blade holder presses on the PDMS substrate and change the slope of the curve in **a**. (**c**) Diagram showing the actual spacing of slits cut on the PDMS versus the designed spacing in the drawing (9 repeats). (**d**) Schematic of the position flexibility of integrated metal microwires. (**e**) Micrograph of integrated metal microwires with angle 0°, 45°, and 80° relative to the channel wall from top to bottom (**f**) Micrograph of integrated metal microwires of length of the width of the channel, 1, 2, 3 mm from top to bottom. (scale bar=1 mm). (**g**) Microwires of different diameter and material inserted into slits. From left to right: 230 μm optical fiber, 125 μm capillary tube, 50 μm copper wire cc 25 μm insulated copper wire. (**h**) A 25 μm micowire written into a slit before sealing. (**i**) A 25 μm microwire written into a slit after sealing (Scale bar=200 μm).

**Figure 3:**
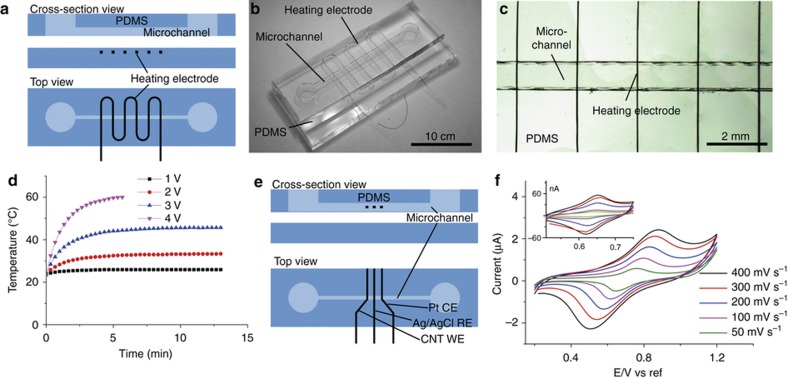
(**a**) Schematic of the PDMS device with integrated heater. (**b**) Photograph of a fabricated PDMS device with integrated heater. (**c**) Micrograph of the integrated heating microwire under the microchannel. (**d**) Controlling the temperature of DI water inside the microchannel by applying different voltage to the heater. (**e**) Schematic of the electrochemical sensor with platinum working, counter electrode and silver/silver chloride reference electrode. (**d**) Cyclic voltammetry of 1 mM ferricyanide with 10 mm electrode under different scan rate. Inset shows results repeating the cyclic voltammetry with 1 mm electrodes.

**Figure 4:**
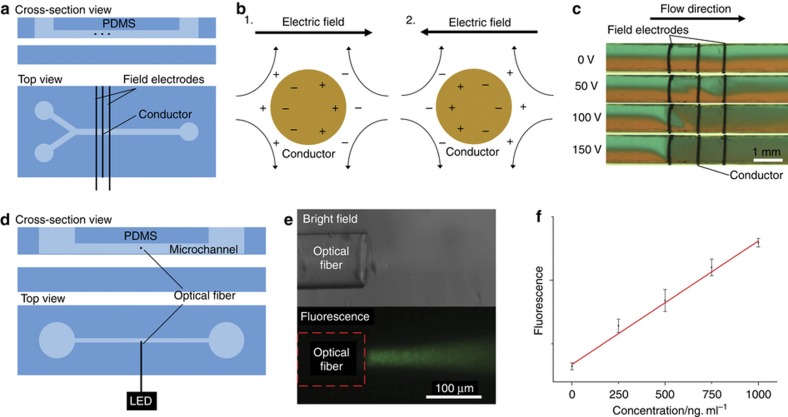
Schematic of the electro-osmosis mixer. (**b**) Principle of electro-osmosis mixing around the metal microwire. 1—electro-osmotic flow around the conductor microwire under positive electric field. 2—electro-osmotic flow around the conductor microwire under negative electric field. (**c**) Mixing under 0, 50, 100, and 150 V applied alternation current (AC) voltage. (**d**) Schematic of the PDMS device with integrated optical waveguide. (**e**) Bright field microscopic image of the integrated optical fiber inside a microfluidic channel (upper) and fluorescence microscopic image of picogreen-dyed DNA excited by blue LED light guided through the optical fiber. (**e**) Relationship between fluorescence intensity and the concentration of the DNA sample.

The xurography PDMS micromolding process is shown in Figure 1a. First, adhesive-backed polyethylene films (Impact-Resistant Slippery UHMW Polyethylene, McMaster-Carr, Inc., Atlanta, GA, USA) with thickness of 127, 305, 559 μm were laminated on the original cutting mat with liner side down. Next, the films were patterned by the cutting plotter (Cricut Explore, Provo Craft & Novelty, Inc., UT, USA) to produce features in the film with the desired microchannel geometry (Figure 1a—1). The unwanted areas were then peeled off with tweezers and the microchannel patterns were left on the cutting mat. Subsequently a transfer tape (Main Tape Company, Inc, NJ, USA) was laminated on the patterns, then gently peeled off together with the microchannel pattern (Figure 1a—2), which was then laminated on a clean silicon wafer (Figure 1a—3). The transfer tape was again peeled off from the wafer to leave the patterns on the wafer. These polyethylene films with microchannel patterns were used as the master mold for the soft lithographic fabrication of the PDMS microchannel. PDMS prepolymer (Sylgard 184, Dow Corning, MI, USA) was prepared by mixing the elastomer base with curing agent in the ratio of 10:1. The prepolymer was poured onto the master mold (Figure 1a-4), degassed under vacuum for half an hour and then cured at 80 °C for 1 h. Then the cured PDMS microchannel was peeled off for electrode integration (Figure 1a-5).

### Electrode integration

The electrode integration process is shown in Figure 1b. First, the slits were cut on the PDMS substrate by the cutting blade of the cutting plotter (Figure 1b—1). The cutting force was set to be between 188 to 620 mN to obtain shallow slits of a defined depth. The PDMS substrate was then laminated onto a 1 mm thick PMMA sheet which was attached to the cutting mat to firmly fix it during the cutting process. Next, the metal microwire was written into the slit using a microwire ‘pen’ (Figure 1b—2). A customized microwire ‘pen’ (Figure 1c) was made by encapsulating the tip of a stainless steel needle (inner diameter 203 μm, 75165A129, McMaster-Carr, Inc., Atlanta, GA, USA) using a polyolefin heat shrink tube (6699T11, McMaster-Carr, Inc., Atlanta, GA). The heat shrink tube prevents abrasive contact of the microwires with the stainless needle tip as the wire slide out and reduces the friction during writing without significantly changing the nozzle diameter (Figure 1d). The customized microwire pen was threaded attached to a 10 ml BD syringe and the microwires were inserted into the pen tip. The microwires were written into the slits manually or automatically by the cutting plotter with two cartridges one of which was used to perform slit cutting and the other to perform microwire writing (Figure 1e).

Next, the PDMS substrate with integrated microwire was coated with uncured PDMS prepolymer as adhesive/sealant and bonded to another PDMS substrate (Figure 1b–3). The slits on the PDMS would potentially cause leakage unless sealed completely. Here the sealing was completed using a wet bonding process^38^. The PDMS prepolymer was prepared by mixing PDMS base: silicone oil (Element 14 PDMS 50-E, Momentive, USA): curing agent to 5:5:1 and then spin-coated on a silicon wafer at 1300 rpm for 30 s. Then the PDMS device with integrated electrodes was stamped on the spin-coated prepolymer layer and placed in a low vacuum environment for 15 min. The prepolymer fills cavities between the wire and the PDMS during this period. Finally, the PDMS prepolymer was cured at 80 °C for half an hour (Figure 1b—4).

## Results and discussion

### Slit patterning

By adjusting the cutting force, different slit depth could be obtained. The depth of the slits is a crucial parameter as it determines the position of the microwire electrodes in the height direction of the microchannel. To find out the relationship between the cutting force and the slit width, arrays of slits were cut on a blank PDMS substrate under various cutting forces. The depth of each slit was measured from images taken of the cross-section of the PDMS substrate with a microscope (S8AP0, Leica, Wetzlar, Germany). Two regimes were identified from the measurements obtained (Figure 2a). In the first regime when the holder of the blade does not contact the PDMS substrate (Figure 2b, left), the slit depth increases linearly with the cutting force with a slope of 2.76. In the second regime where the holder comes in contact with the substrate (Figure 2b, right), it compresses the substrate and the depth of cut increases linearly with a reduced slope of 1.39. The minimum depth for this cutting machine is around 150 μm. Further lowering the force increases the variability of the depth of cut. The same experiment was conducted with another cutting plotter—Silhouette SD cutting plotter (QuicKutz, Inc., Lindon, UT, USA). The adjustable working length of the blade in the Silhouette cutting plotters make it possible to cut slits with shallow depth down to 33 μm.

The minimum spacing achievable between two slits was also characterized (Figure 2c). Arrays of slits with varied spacing between them were cut on blank PDMS substrates and observed under a microscope. The actual spacing measured with a microscope showed repeatable slit spacing down to 150 μm. Higher precision cutting plotter^35^ that can accurately position the cutting blade and increasing curing agent ratio to 5:1 (Ref. 37) that produces stiffer PDMS might improve the minimum spacing resolution.

As shown in Figures 2a and c, the microwire electrodes can be written into microelectrode patterns with spacing resolution down to 150 μm and the position of the microelectrode pattern in the height direction can be adjusted as well. Therefore, this xurographic method allows grooves suitable for insertion of microwires at a variety of locations and positions inside the microfluidic device. As shown in Figure 2d, the microwire electrodes can be written behind the microchannel sidewall (blue), inside the microchannel (red) or under/upon the microchannel (yellow) with adjustable distance. This kind of 3D positioning flexibility and simultaneous integration with microchannels is not possible in other microelectrode integration methods. Even though extrusion-based 3D printing has been recently used to form 3D microelectrode structures using viscous silver ink^39^, they are suitable for free standing structures such as inductors and coils but are difficult to integrate inside and across microfluidic channels where leak proof sealing and bonding considerations are important.

### Microwire electrode writing

When the microwires were inserted into slits in PDMS, they are held in place by the friction between the microwires and PDMS^40^. Pipette tips, stainless steel needles, and plastic needles were tested to find a suitable ‘pen’ to write the microwires into the slits. The tip size of a plastic pipette tips (AD10EK, Diamed Lab Supplies Inc., ON, Canada) was found to be too large (inner diameter of 800 μm) to be inserted into the slit and to write microwires into patterns with spacing smaller than the outer diameter of the tip. Plastic needles with 200 μm inner diameter (6699A8, McMaster-Carr, Inc., Atlanta, GA, USA) were small enough to insert into the slit during writing but their tips bent easily due to insufficient strength. The stainless steel needle with 200 μm inner diameter (75165A128, McMaster-Carr, Inc., Atlanta, GA, USA) can be easily inserted into the slit without bending. However, the friction between the needle and the electrode microwire was high enough to drag the microwire out of the slit or even break the microwire. To lower this friction, a miniature heat-shrink tube was sleeved on the stainless needle tip. The length of the heat-shrink tube was about 500 μm longer than that of the needle tip. The heat shrink tube was then heated with a heat gun until fully-shrunk. The extra length of the heat shrink tube shrank to about the same size as the stainless steel needle tip (Figure 1d). Due to this extension the microwire primarily contacted the polymeric heat shrink material as it emerged out of the needle, reducing the high frictional contact with stainless steel. This enabled the wires to flow smoothly out of the tip and be held by the PDMS without breaking.

Most of the microwire electrodes are written into slits and the electrode pattern was defined by the slit pattern. However, when the microwires were written into the channel, there was no "guiding" slit to define the electrode pattern. Instead, the electrode pattern inside the channel was controlled by the slit position at the channel wall. It was demonstrated that the angle (Figure 2e) and the length (Figure 2f) of the electrodes could be locally controlled within the channel by appropriately designing the slit pattern. The angle of the electrode can be adjusted to obtain desired electric/magnetic field direction and the length of the electrodes can be adjusted to obtain desire electrode surface area.

Different diameter microwires of different material was also tested for their suitability to be written into patterned slits. Optical fiber (250 μm), capillary tube (125 μm), bare copper microwire (50 μm) and insulated copper microwire (25 μm) were written into 500 μm slits and imaged with scanning electron microscope (Figure 2g). The integration of optical fiber demonstrated that not only electrodes but other components in the microwire form could be integrated.

### Device bonding and sealing

Oxygen plasma bonding is a widely used irreversible bonding method for PDMS. However, initial devices fabricated using oxygen plasma bonding, leaked around the microwire electrodes written as there were gaps formed between the slit and the wire that were not sealed. Therefore a modified wet bonding process^38^ was used. A PDMS prepolymer layer was spin-coated on a silicon wafer and transferred to the PDMS substrate by contact printing. Another PDMS substrate was then aligned and bonded together. First, 10:1 PDMS prepolymer was used which resulted in partial sealing and prevented leakage under normal operation but not under high pressure. To better fill the gaps with the prepolymer, silicone oil was added to uncured PDMS prepolymer to lower its viscosity. Unlike organic solvents that are commonly used to lower the viscosity of PDMS, the silicone oil is not volatile and remains in the cross linked matrix and is more robust in preventing leakages^41^. The ratio of the silicone oil to PDMS base was increased from 1:10 to 5:5 until sealant penetrates all the crevices as observed under microscope. The device was vacuumed for 15 min to further improve the sealing efficiency. SEM image showed that the gap around the integrated microwire (Figure 2h) was completely sealed (Figure 2i) after wet bonding with the modified PDMS prepolymer. The bonding strength was measured by burst test with bonded device with and without integrated microwires. The burst pressure for the wet bonding of the PDMS device without integrated microwire went up to 517 kPa while that of the PDMS device with integrated the microwire was able to withstand up to 310 kPa.

### Applications

In order to demonstrate the versatility of this method for integration, a heater, an electrochemical sensor, a electrokinetic mixer and an optical waveguide were integrated into PDMS devices and characterized.

### Heater

Heaters are a key component of devices requiring temperature control such as those used in DNA amplification^42^, thermally controlled micro-valve^43^ and as microreactors^44^. These heating microwires should be insulated from the contents of the microchannels. The process flow for conventional fabrication of planar microheaters requires additional steps to deposit and pattern the insulation layer on top of the heater microwires^43,45^.

Here a 50 μm Ni–Cr microwire heater was integrated directly into the bulk of a flat PDMS substrate and then bonded to another PDMS layer with microchannel structure (Figures 3a and b). The spacing of the heater microwire pattern is 2 mm (Figure 3c). The distance between the heater and the channel could also be controlled by the depth of the slit. To test the fabricated heater, DI water was injected into the microchannel. Voltage from 1 to 4 V was applied to the heating microwire. A thermocouple was inserted into the middle of the microchannel from the reservoir to monitor the temperature inside the channel (Figure 3d). Temperatures in the range of 20–60 °C can be easily achieved using these heaters. The higher the voltage applied, the faster the temperature rise and the higher the equilibrium temperature achieved. Higher applied voltages or tighter windings can allow higher temperatures to be reached faster.

With the xurographic method, heater wires can be directly integrated underneath the microchannels thereby insulating them from the contents of the channel. Conventional fabrication of microheaters would require an additional fabrication step to deposit the insulation. Alternatively, insulated copper microwires (which are commercially available) can be written directly into the channel which will provide fast heating and good electrical insulation.

### Electrochemical sensor

Electrochemical sensors are increasingly popular in microfluidic devices because they are easy to be miniaturized^46^. Unlike the heater example, electrodes for electrochemical sensing should be in direct contact with the samples in the microchannel. Almost all the popular electrode materials for electrochemical sensing including gold, platinum, carbon fiber are available in microwire form. A 3-electrode electrochemical sensor was fabricated to demonstrate that our method is capable of integrating these electrodes into the middle of a microchannel (Figure 3e). It has a 15 μm carbon nanotube microwire as working electrode, a 50 μm platinum microwires as the counter electrode and a 50 μm silver/silver chloride microwire as reference electrode. These wires were sequentially inserted into the respective slots cut previously using the xurography process described in methods section. Silver chloride reference electrode was made by electrochemically growing silver chloride on silver microwire surface^47^. Briefly, the device was filled with 1 M KCl solution and then 2 V DC anodization was applied to grow silver chloride on the silver microwire electrode. The electrode surface area was determined by the length of electrode exposed in the channel which can be controlled to using the electrode insertion schemes described in previous sections. Sensors with 1 and 10 mm electrode were made and tested by performing cyclic voltammetry with 1 mM Potassium Ferricyanide in 1 M KCl using potentiostat (Emstat, PalmSens Inc., Houten, Netherlands). The cyclic voltammetry was run between 0.2 to 1.2 V for 10 mm electrode and 0.5 to 0.8 V for 1 mm electrode with step voltage of 5 mV at varied scan rate (Figure 3f). The experiment was repeated 10 times for each scan rate with similar results. The signal obtained from the device with 10 mm electrode is much higher than that from the device with 1 mm electrode.

The xurographic method enabled the integration of high quality electrodes of multiple materials (carbon, platinum and sliver) very easily and within a few mins at precise locations into the microfluidic devices. Conventional processing will require multiple processing steps which will take long duration of time for achieving the same multimaterial integration. Furthermore, the positioning of the sensing electrode in the middle of the flow channel rather than at the bottom also enables vortex shedding even at low Reynolds number (>40) which can facilitate better sensing of trace analytes in the sample fluid. This will be important in a variety of applications including biosensing and environmental sensing.

### Electrokinetic mixer

Mixing is a challenging issue in microfluidics due to the low Reynolds number inside the microfluidic channel. Induced charge electro-osmosis^48^ (ICEO) has been widely investigated as an effective method for microfluidic pumping^49^ and mixing^50^. Here a ICEO mixer using the new fabrication technique was designed with three parallel 50 μm copper microwires, 1 mm apart from each other (Figure 4a). The 10 kHz AC from 0 to 200 V was applied to the microwires at both ends to generate strong electric field from 0 to 1000 V cm^−1^ in between. The middle microwire subject to the electric field polarizes and drives electro-osmotic flow around it (Figure 4b). This ICEO setup ensured that the electric field was applied only in a local region between the two microwires and the mixing effect was also confined. Deionized water dyed with different food coloring was pumped into the two inlet channels at 5 μL min^−1^ and the mixing was observed under the microscope (Figure 4c). The fluids with different color mixed around the middle microwire and tend to be fully mixed at higher electric field strength. These results indicate that a compact active micromixer can be integrated using these microwires.

In conventional electrokinetic mixers^49,51^, the electrokinetic flows are generated on the microwire surface in the bottom of the microchannels and have limited effect on the bulk solution. Integrating the metal microwire into the middle of the microchannel could increase the mixing efficiency by disturbing the flow inside the bulk solution.

### Optical waveguide

Even though electrochemical sensors are widely used in LOC devices, they are still have limitations including requirement for electroactive enzyme labeling and sensitivity to temperature and pH^52^. Due to these limitations, optical detection is still preferred in some areas like genomic sensing. Integrating waveguide, filters and other optic components into LOC devices can reduce cost and enhance sensitivity. Here we demonstrate that our method can be used to integrate these optical fibers into PDMS microfluidic devices. In this application, the optical fiber is used as a waveguide to route the excitation light to a local spot inside a microfluidic device in order to fluorescently measure DNA concentration. The tip of a 250 μm optical fiber (FG105UCA, Thorlabs, Newton, NJ, USA) was striped (over a length of 10 mm) to remove of the protective coating. The striped length of the fiber was then reduced down to 1–2 mm by cleaving the extra length. The fiber prepared in this way is stable and does not break during writing because most of the fiber is protected by the coating. The optical fiber was written into the desired position of the channel with a pipette tip (AD10EK, Diamed Lab Supplies Inc., Mississauga, ON, Canada) due to its relatively large diameter (Figure 4d). DNA samples with different concentration from 1 ng ml^−1^ to 1 μg/ml was dyed with picogreen and injected into the microchannel. Excitation light of 490 nm generated by a fiber-coupled LED (M490F2, Thorlabs, Newton, NJ, USA) was guided through the optical fiber into the microchannel. The emission light was detected with fluorescence microscope (Figure 4e). The fluorescence intensity was obtained by the mean brightness of a 100 μm×100 μm square area in the middle of the beam. The fluorescence intensity increased linearly with increased DNA concentration (Figure 4f), which is consistent with the quantitation curve of picogreen reagent^53^. Using optical fiber the excitation light could be routed in a orthogonal direction to the detector simplifying the detection process.

## Conclusions

A modified xurographic method to integrate metal microwire into PDMS microfluidic devices based on xurography has been reported. The microchannel fabrication and metal wire integration were incorporated into a low-cost (US$200) cutting plotter^54^ to provide a complete xurographic lab on chip fabrication solution. No expensive equipment, clean room environment, time consuming and labor intensive operation was involved.

Metal microwires down to 15 μm were successfully integrated at various locations into the microchannel structure providing an unprecedented 2.5 D control over its location (2D pattern with adjustable position at the height direction). This feature was demonstrated by integrating metal microwire suspended inside the middle of the microchannel. Various capabilities of the xurographic process was characterized. Microfluidic heater, electrochemical sensor, electro-osmotic mixer and optical waveguide were integrated into PDMS microfluidic devices to demonstrate the versatility of the modified xurographic method. This low-cost method to integrate high quality microwires will be of widespread interest and will find use in a number of microfluidic applications requiring integration of electrical/optical sensors, heating/cooling elements or actuators into microfluidic devices.
